# Using participatory research to co-produce an education and training e-resource to support care home staff to meet the sexuality, intimacy and relationship needs of care home residents with and without dementia

**DOI:** 10.3389/frdem.2023.1235517

**Published:** 2023-12-11

**Authors:** Maria Horne, Jane Youell, Christine Brown Wilson, Laura Brown, Paul Simpson, Tommy Dickinson

**Affiliations:** ^1^Faculty of Medicine and Health, School of Healthcare, University of Leeds, Leeds, United Kingdom; ^2^School of Nursing and Midwifery, Queens University, Belfast, United Kingdom; ^3^Division of Psychology and Mental Health, School of Health Sciences, University of Manchester, Manchester, United Kingdom; ^4^School of Social Sciences, University of Manchester, Manchester, United Kingdom; ^5^Department of Mental Health Nursing, Kings College London, London, United Kingdom

**Keywords:** sexuality, intimacy, relationships, education and training, aged care, community-based participatory research

## Abstract

**Aim:**

To present the methodological approach and research methods chosen in a research study designed to enable the collaborative creation of an education and training e-resource designed to facilitate and support care home staff to address the sexuality, intimacy and relationship needs of older care home residents.

**Design:**

Co-production using community-based participatory approach.

**Methods:**

Four participatory workshops with care home staff, residents and their significant others.

**Results:**

Workshops 1 and 2 identified and developed real-world case scenarios related to sexuality, intimacy and relationship needs and identified care staff training needs in this area. Then workshop 3 provided valuable feedback on the prototype training e-resource, and the final workshop identified care home staff engagement with and implementation of the e-resource in practice.

**Conclusion:**

The findings provide evidence that using participatory approaches, such as co-production, to develop education and training resources in a sensitive subject area with care home residents, significant others, carers and care home staff, was a useful approach in engaging a vulnerable population group, in a sensitive area. However, this approach is not without challenges in care home communities.

## 1 Introduction

Sexuality, intimacy and relationships remain important into old age (Thorpe et al., 2015; Fileborn et al., 2017). However, barriers to enabling expression of sexuality, intimacy and relationships needs in residential care facilities, holistic living environments that provide essential long-term care and support tailored to the unique needs of individuals, have been identified. Frailty, policy, and lack of privacy have also been found to restrict the expression and support of fulfilling sexual, intimate and relational needs (Bauer et al., 2013; Villar et al., 2014). This paper focuses on care homes. These are collective living environments where nursing, and/or personal care, and accommodation are provided together [Social Care Institute for Excellence (SCIE, 2021)]. Residents suggest that tolerance and knowledge of sexuality, intimacy and relationship needs in older age are low among nursing care home staff, that medication has a negative impact on sexual expression and that the lack of available suitors, particularly for heterosexual women, is a significant barrier (Villar et al., 2014). Care staff attitudes can also present a further barrier, with a lack of understanding of the importance of sexual expression, and with sexual expression being considered irrelevant or even disruptive in residential care (Ward et al., 2005). A lack of knowledge and understanding, and the presence of prejudice, ageism, and judgmental attitudes on the part of care staff have also been identified as major barriers to supporting residents needs with calls for more education and training to raise awareness and address these issues (Bauer et al., 2013; Simpson et al., 2017b). In an earlier audit (Brown et al., 2018) care home staff identified the importance of education and training in this area and that the views of care home staff, significant others and care home residents should be included. Co-production and co-design are established methods used for co-creation of knowledge in the public sector (Alford, 2014). Co-production has been used effectively in health and care service delivery (NIHR, 2021) and in the development of health care training resources (Tripney and Powel, 2016; Patel et al., 2017). Co-production and co-design are terms often used interchangeably in the literature (Voorberg et al., 2015). Brandsen and Honingh (2015) suggest a typology of co-production where multiple stakeholders are engaged at different levels of a service including implementation as well as co-design. Indeed, the term co-production could be considered as an umbrella term with co-design an example of an activity when using co-production methodology (SCIE, 2013; NIHR, 2021). Therefore, this study aimed to co-produce an interactive education and training e-resource to support the sexuality, intimacy and relationship needs of older residents living in care homes through a community-based participatory research approach.

### 1.1 Underpinning theoretical frameworks and approaches

Community-based participatory research (CBPR) (Minkler and Wallerstein, 2008) is a partnership approach to research that directly seeks to engage community members in sharing their knowledge and perspectives (Israel et al., 1998). CBPR is generally considered to be a form of participatory action research (McIntyre, 2007) that is collaborative between researchers and community members, a cooperative enterprise (Green and Thorogood, 2005) that engages communities, as active and equal partners, in the design and implementation of research that benefits the intended community (Israel et al., 2013) and aims to reduce inequalities in health and wellbeing (Baum et al., 2006). CBPR approach has eight core principles and values: (i) a recognition of the community as a unit of identity; (ii) builds on the strengths and resources within the community; (iii) facilitates collaborative partnerships in all stages of the research; (iv) integrates knowledge and action for mutual benefit of those involved; (v) promotion of a co-learning and empowering process that addresses social inequalities; (vi) involves a cyclical and iterative process; (vii) addresses health from both positive and ecological perspectives and (viii) disseminates findings and knowledge gained to all involved (Israel et al., 1998).

Although notoriously difficult to define, the term community is now generally described more broadly than geographical location or group, as a multi-faceted concept including diversity, social ties, shared viewpoints and engaging in joint action in various settings (MacQueen et al., 2001). Public and policy frameworks primarily focus on geographical location with little emphasis on social relations and experience (Walkerdine and Studdert, 2016). The methodology utilized within this research study aimed to acknowledge these differing perspectives by creating a research team that included social psychologists, educationalists, nursing professionals and sociologists. The purpose of this collaborative research study was to include the views and experiences of differing residential community participants—care home staff, care home residents and their significant others about the provision of opportunities and support for meeting the sexuality, intimacy and relationship needs of older adults and how care home staff could provide support these needs through the development of a tailored education and training e-resource. Therefore, for this study, we defined “*community based*” as a community of place, interest and/or identity. Using CBPR approach was deemed to be a more engaging, facilitative, and supportive approach to undertaking research in this sensitive area of need with a vulnerable population group.

Participatory research approaches seek to increase knowledge whilst acting practically to bring about positive change (Jensen and Laurie, 2016). Increasingly, participatory research approaches have been used in health research to conduct studies with key stakeholders, including academics, policymakers, healthcare staff, patients and/or service users. The ethos of this approach is to conduct research *with* not *on* or *about* participants (ICPHR, 2013). Whilst community-based participatory research moves away from the traditionalist “*outside expert*” approach, it should be acknowledged that this approach is often challenging. Working in collaboration with communities to bring about social change requires consideration to be given about what constitutes a community; how to involve community members in the research study; how to manage conflicts of interest; what is considered to be social change and also consider if the research timescales respond to perceived community needs (Nigro, 2017). These issues are often difficult to navigate (Nigro, 2017).

Researchers and communities must negotiate the research process together, navigating through power dynamics, individual agendas, methodological and ethical considerations within the broader societal context (Minkler and Wallerstein, 2008). However, actively engaging end-users in the research process allows the research design to be more responsive to the needs of the community as it is grounded in the diverse perspectives and expertise of both researchers and community members and other key stakeholders (Elg et al., 2012). Such co-creation of knowledge may also allow for tailoring of interventions to manifest more effectively than traditional top-down approaches (Elg et al., 2012).

The benefits of CBPR approaches are well-documented (Brush et al., 2020) and research funders actively encourage community engagement in health and healthcare research (NIHR, 2022), as it is recognized that patient and public involvement can positively impact research. Almost half a million people live in care homes in the UK (Carehome.co.uk, 2023) and around 70% of all care home residents are reported to have dementia or severe memory problems (Alzheimer's Society, 2023). Therefore, care home resident engagement and involvement in research is important in shaping their care experience. However, the majority of the CBPR literature reports on studies conducted with community-based organizations (Strike et al., 2016). Few studies report on improving health service delivery for persons with dementia and their caregivers in rural and remote settings (Morgan et al., 2014) and in end-of-life care (Williams et al., 2005). Even fewer studies report on using this approach in hospital (Strike et al., 2016) and care home settings despite older care home residents being under-represented in research (Backhouse et al., 2016). Regardless of this under representation, some small-scale research studies have managed successfully to engage with older residents (Shura et al., 2010; Chenoweth and Kilstoff, 2018). For example, Shura et al. (2010) and Chenoweth and Kilstoff (2018) found that involving older care home residents in a participatory research approach brought about effective organizational change and created a sense of social cohesion between residents. There is little evidence that family members of care home residents have been included in community-based participatory research studies. However, Hewitt et al. (2013) have acknowledged that care home environments are made up of residents, staff, and the broader local community, including participants from each group in their quality-of-life study for older care home residents. Interestingly, community based participatory research studies which do involve family members focus on end-of-life care, arguably as difficult a topic for discussion as sexuality and intimacy in older people's residential care. Caswell et al. (2019) successfully engaged bereaved carers, care workers and third-sector stakeholders to co-design and co-create a training package aimed at supporting at home end of life care, whereas Stone et al. (2013) recruited participants from the care home community, their relatives and staff to participate in a qualitative study based on engagement with Advance Care Planning. The findings around end-of-life care mirror many of the findings around sexuality and intimacy, with staff needing knowledge, skills and confidence to discuss advance care planning and end-of-life wishes.

Co-production has been used effectively in health and care service delivery (NIHR, 2021) and in the development of health care training resources (Tripney and Powel, 2016; Patel et al., 2017). For instance, Patel et al. (2017) co-created an oral health training program for care home staff and carers to improve the oral health of residents. Care home resident involvement included informal discussions and observation of oral care practice. Similarly, Tripney and Powel (2016) describe engaging healthcare support workers, to include their voice on identifying training needs, through the creation of an advisory board. By engaging healthcare support workers using a co-production approach the training resources were identified by the workforce and subsequently evaluated positively suggesting they were more appropriate and relevant for the specific needs of the healthcare support workforce (those most likely to receive training). Co-production was found to stimulate change and urgency based on patient and staff involvement (Vennik et al., 2016).

Despite the benefits of utilizing such an approach, the use of co-production may bring challenges due to the diversity of stakeholders involved resulting in competing demands and viewpoints (Alford, 2014; Palumbo and Manna, 2018). To manage competing viewpoints, various approaches/methods have been proposed. For example, the use of consensus meetings to enable competing viewpoints from multiple stakeholders to be heard by using consensus-based activities such as “*sticky walls*” (Brown Wilson and Slade, 2018). Whilst others created alternative spaces for dialogue to take place recognizing that co-production is also contextual (Sapouna, 2021). Tembo et al. (2021) argued that resistance in co-production can be addressed by ensuring that feedback to the communities involved should be more creative, citing the use of plays, puppet shows or comics. Others have suggested the use of a bricolage approach as a technique to encourage stakeholder engagement (Melville-Richards et al., 2020).

Creative techniques, specifically storyboarding, have been found to benefit healthcare education (Dexter, 2016). This is a narrative pedagogic tool that has been positively evaluated in nurse education (Lillyman et al., 2011) and health research (Lupton and Leahy, 2019). Various materials are provided, such as large sheets of paper, pens, pencils, post-it notes, etc. There are also various ways in which this might be undertaken; individually, by encouraging group members to each complete a storyboard or in a group facilitated by a researcher using words or pictures (Redman-MacLaren et al., 2014; Cross and Warwick-Booth, 2016). The sticky wall method, a way of collecting qualitative feedback, is one variation which captures thoughts and ideas which come from discussion. This method involves participants writing ideas and comments on sticky notes which are displayed on the wall. Ideally, the group then works to cluster the sticky notes into themes. The advantage of this method is that sticky notes can be reallocated as discussion continues and broader themes emerge. The outcome of the creation of a sticky wall can be photographed for further analysis. Sticky walls have been found to offer a time-efficient method of gaining feedback (Evaluation Support Scotland, 2019).

### 1.2 Aim

The overall aim of the research study was to co-produce an education and training e-resource through a participatory approach applied in care home settings with key stakeholders—care home staff, care home residents and their significant others i.e., relatives/loved ones/spouses/partners, that could facilitate and support care home staff to engage with and support the sexuality, intimacy and relationship needs of care home residents. In this paper we present the methodological approach and research methods chosen to enable the collaborative creation of the aforementioned education and training e-resource based on this research study.

## 2 Methods

### 2.1 Team and advisory group input

Guided by principles of CBPR approach (Minkler and Wallerstein, 2008), an advisory group was convened with members made up of lay representatives, experts in safeguarding, LGBTI+ communities, Patient and Public Involvement, the law and aging and sexuality. Advisory group meetings occurred three times across the course of the first year of the study that the research team attended. The research team comprised of six academics with specialist knowledge of care home environments, public health, aging and sexuality, sexuality and dementia, who all had teaching and research experience. In addition to advisory group meetings, the research team held meetings via Zoom approximately every 2 months for the duration of the study. By creating such diverse involvement, pedagogy, practice and policy were equally considered and each group member played to their individual expertise to inform the design and development of the education and training e-resource.

### 2.2 Design

Stakeholders were invited to facilitated workshops, guided by the following questions:

- What are the current issues facing care home staff, residents, and significant others in connection to the sexuality, intimacy and relationship needs of older people living in care homes?
- What are care home staff knowledge gaps around sexuality, intimacy and relationship needs of older people living in care homes?
- What strategies can be identified to enable care homes to utilize any education and training e-resource developed?

Each workshop employed creative techniques and consensus methods, such as sticky walls and storyboarding to ensure all perspectives were gathered and informed the final resource.

Ethical approval was granted by the University of Leeds, School of Healthcare Research Ethics Committee. As sexuality and intimacy is a sensitive subject, a distress protocol was in place to support participants should they experience distress during the workshops. Potential safeguarding concerns were considered and the appropriate contact details for local safeguarding teams were made known to the researchers, should this be required. Further consideration was given to the power dynamics between care home staff, care home residents and their significant others, with each group invited to attend separate workshops, to ensure that care home resident voices were heard and valued along with those of relatives/loved ones/spouses/partners and care home staff.

### 2.3 Participants and recruitment

Recruitment commenced in December 2018 and ended in June 2019. Potential care homes were identified via the Enabling Research in Care Homes (ENRICH) Research Network and sent an information pack, including invitation letter, information sheet, researcher permission to contact the care home manager form and a postage paid envelope. Care home staff and family members and/or significant others, who provided insight into accounts of dementia and intimacy, were recruited through the care home manager via staff meetings and advertisement of the study at the participating care homes. Care home residents, who were aged 60 years and over, residing in a care home and having capacity to consent, were purposively recruited via the care home managers.

### 2.4 Data collection

**Figure 1:**
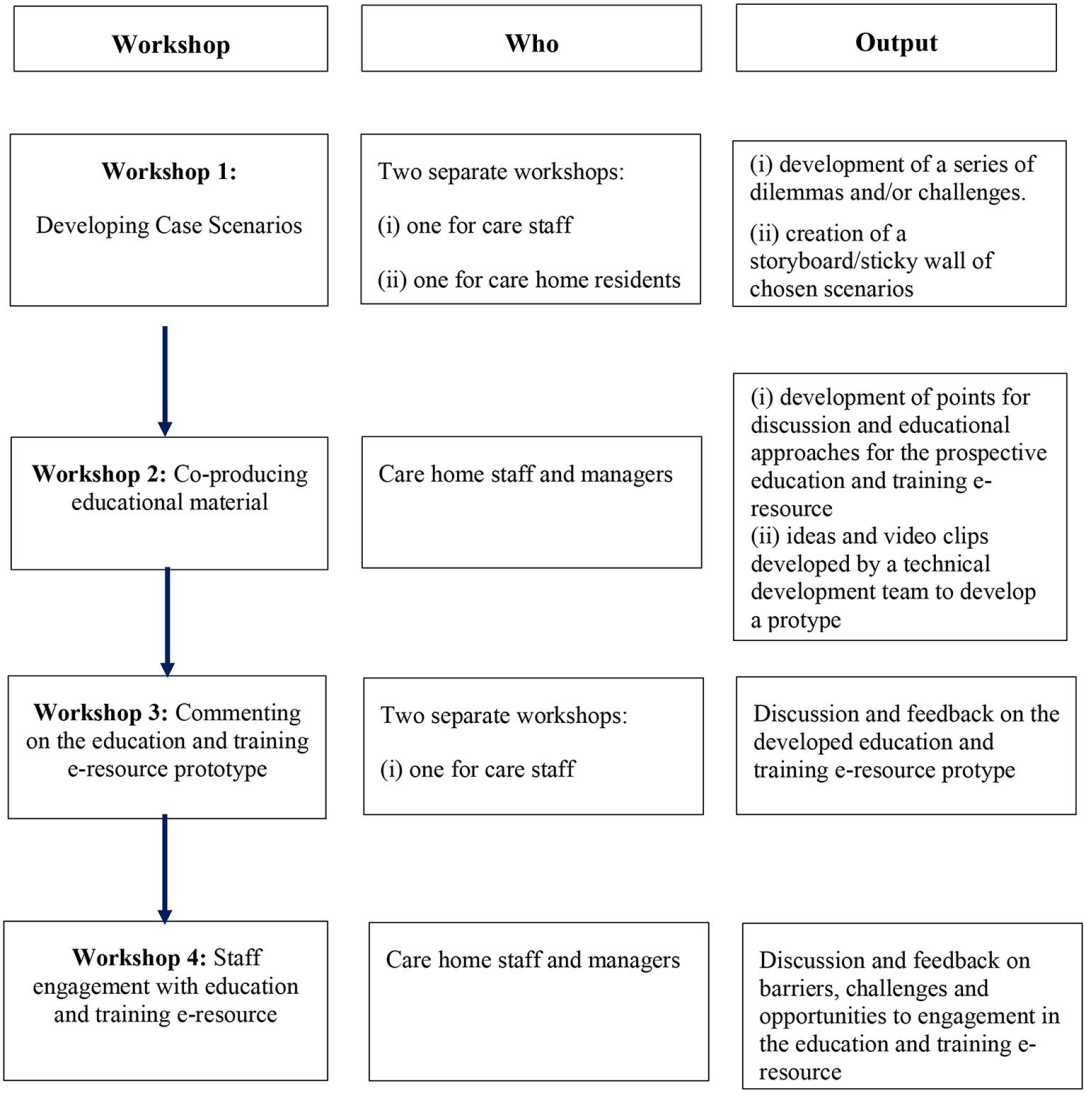
Flow chart of the workshops, participant groups involved and output.

Four workshops (see Figure 1) were held as follows.

#### 2.4.1 Workshop 1: developing case scenarios

Separate workshops were held with nursing care home staff and managers and then with residents and their relative/spouse/significant other. The purpose of workshop 1 was to develop real-world case scenarios in discussion with care home staff, care home residents and their significant others.

Group facilitation was guided by the literature/materials identified through a scoping review (Horne et al., 2021), stimulus material (vignettes/case scenarios) from our previous work (Simpson et al., 2018) was also used to prompt discussion. Specifically, care home staff and managers, residents and their significant others were asked: (i) for feedback on given real-world scenarios (to prompt discussion) and asked to provide stories/scenarios they would be willing to share to develop real-world case scenarios based on their experience (“do you have other stories you are happy to share with us?”) and (ii) asked to watch some short videos about sexuality, intimacy and relationship needs of care home residents (Aged Care Awareness, 2019) and to provide comment on the length, content and language used to inform development of the subsequent education and training e-resource. To aid the sharing of practice and/or personal experiences, participants were invited to create sticky walls, using post-it notes, to facilitate this process.

Formal qualitative content analysis of the identified themes/scenarios from this workshop was then undertaken and presented to staff in Workshop 2.

#### 2.4.2 Workshop 2: developing educational material

Workshop 2 only included nursing care home staff and managers. The purpose of workshop 2 was to discuss with care staff the initial themes identified from workshop 1 and to explore and identify educational needs relating to each theme. This workshop used story boards to develop points for discussion to advance educational material for the subsequent education and training e-resource. The same stimulus materials (vignettes/case scenarios) were used to prompt discussion. Participants were asked to contribute their thoughts and given prompts such as: “What do you think about the given scenarios?” and “how might you approach the given scenarios?” The results of this workshop were intended to inform the subsequent education and training e-resource development and display, for example content/appearance/presentation of the education and training e-resource.

The research team checked with care staff whether the developed real-world case scenarios identified within each theme related to their practice and represented authentic examples. Using these real-world examples as a case study, care home staff discussed their learning needs around each scenario with discussions being captured using the sticky wall method. Care home staff also commented on education and training in general and highlighted likes and dislikes.

The findings from this workshop influenced and informed the development of a prototype education and training e-resource.

#### 2.4.3 Workshop 3: what do you think of the prototype?

Workshop 3 included both care home staff and managers and residents and their significant others **in separate groups**. A prototype of the education and training e-resource was developed from workshops 1 and 2 and brought back to the same group of nursing care home staff and managers, care home residents and their significant others to try out, elicit further discussion and provide further feedback to refine the education and training e-resource prototype.

#### 2.4.4 Workshop 4: how can we get staff to engage with this?

Workshop 4 was for nursing care home staff and managers only. The final version of the interactive education and training e-resource was shared with care home staff for their feedback. Discussion also focused on how best to encourage care home staff to engage with the education and training e-resource. Barriers, challenges and opportunities around feasibility, acceptability, engagement and implementation were also explored to assist with future implementation of the education and training e-resource.

All workshops were audio-recorded with participants consent and field notes were written after each session.

### 2.5 Analysis

Data produced during the workshops were analyzed using qualitative content analysis (Mayring, 2000). At each workshop, the discussions captured using the sticky wall method were summarized through initial content analysis with the participants and subsequently the text of the workshops were then reviewed by two researchers (MH and JY) to gain understanding and develop themes for units of learning for the educational resource. This was then reviewed by the research team and advisory group and then subsequently fed back into the next workshop for participants feedback and views. Therefore, in terms of dependability, the themes were checked by participants for accuracy. In terms of credibility, the interpretation—the education and training e-resource prototype, was presented to participants to check for authenticity and relevance. To ensure the end-product was transferable, real work scenarios developed from care home staff, care home residents and their significant others were used for the education and training e-resource modules.

## 3 Results

Care home managers recruited staff (*n* = 20), care home residents (*n* = 8) and significant others (relatives/loved ones/spouses/partners) (*n* = 5) using the advertising materials supplied by the research team. Table 1 provides further details of the participating care homes.

**Table 1:** Participating care home summary information.

	**Type of registered care home**	**Age criteria for admission**	**No. of beds/rooms**	^*^Care Quality Commission (2018) rating (at time of study)
Care home: 1	Old age	Ages 65+	40	Requires improvement
Care home: 2	Dementia, learning disability, mental health, old age, physical disability, sensory impairment	Ages 65+	30	Requires improvement
Care home: 3	Dementia, old age, physical disability, sensory impairment	Ages 65+	96	Requires improvement
Care home: 4	Dementia, old age	Ages 55+	50	Good

^*^Care Quality Commission, 2018. Key lines of enquiry for healthcare services. Available from: https://www.cqc.org.uk/guidance-providers/healthcare/key-lines-enquiry-healthcare-services (accessed August 22, 2023).

### 3.1 Participants

In total, thirty-three participants were recruited across four care homes in West Yorkshire, England. Twenty staff members volunteered to take part in the project. All identified themselves as white British. The range of care home staff involvement across roles is detailed in Table 2, showing commitment from the management of the participating care homes and involvement with high proportion of direct care staff.

**Table 2:** Care home staff demographic characteristics (*n* = 20).

**Participant details**	**Number**
**Role**	
Manager	2
Deputy manager	4
Care worker/senior care assistant	10
Care team leader	1
Kitchen staff	1
Administrators	2
**Ethnicity**	
White British	20
Age range	26–59
**Gender**	
Female	19
Male	1
**Sexual identity**	
Heterosexual	18
Gay man	1
Gay woman/lesbian	1

Eight care home residents were recruited across three of the four care homes through their respective care home managers. Five care home residents were female, three male, ranging in age from 60–90 years. All identified themselves as white British and heterosexual.

Five family members were recruited from only one of the four care homes through the care home manager and advertisement of the study at the care home. All were female and were the daughters or daughter-in-law of a female care home resident. All identified themselves as white British, heterosexual and ranged in age from 51 to 64 years.

Care home resident recruitment was low across the care homes. Reflecting on our methodological choices, it was felt that group discussions might have precluded open discussion. Therefore, to secure confidentiality, individual interviews were offered to care home residents as an alternative means of participation, which resulted in further resident recruitment. During one-to-one interviews, residents showed reluctance to engage in the sticky wall method and preferred to discuss their own experiences. Only one couple was recruited to the resident workshop. This couple were interviewed together during workshop 1. A further one-to-one care home resident interview took place.

### 3.2 Creating a collaborative environment

Four workshops were facilitated in four care homes in the West Yorkshire, England. Participants discussed and mapped out their past experiences, dilemmas around sexuality, intimacy and relationship of older care home residents during the facilitated, co-productive workshops.

In one care home a workshop with five care home residents was facilitated. Due to mobility issues and space within the care home, the sticky wall method was not practical. Instead, the views of the participants were captured by the facilitator who made notes as participants contributed to the discussion and posted post-it notes on the wall on their behalf to capture and place their views. The views of these residents contributed to the further development of the education and training e-resource from a resident perspective.

One care home recruited five relatives (all daughters or daughters-in-law of residents). The use of a sticky wall method was acceptable, but there was greater reliance on the workshop facilitator to capture and place their views. Photographs of the sticky walls were taken to provide accurate records for further analysis.

Care home staff attendance was consistent across the four workshops which is due largely to the keenness of care staff to influence this under researched area. Care home residents and their significant others were invited to two of the four workshops, workshops 1 and 3, in order to help identify key topics to be covered in the training and to offer feedback on the prototype (Table 3).

**Table 3:** Participant numbers by workshop (WS).

**Care home**	**Total participants**	**WS1 staff**	**WS2 staff**	**WS3 staff**	**WS4 staff**	**WS1 residents and significant others**	^*^WS2 residents and significant others	**WS3 residents and significant others**	^*^WS4 residents and significant others
1	8	4	3	2	0	Five residents	–	0	–
2	15	6	7	5	4	Five significant others	–	Three significant others	–
3	7	4	4	2	4	Two residents	–	One resident (interview)	–
4	3	3	3	3	0	0	–	0	–

^*^Residents and significant others were not invited to workshops 2 and 4.

### 3.3 Workshop 1: co-producing case scenarios

Workshop 1 lasted for between 60 and 90 min. For care home residents addressing intimacy needs as opposed to sexuality was important. One resident reported having a physical relationship with another resident but that this caused problems with family members.

Some family members reported that they liked staff to show affection to their relative, whist other family members expressed concerns around safeguarding issues, particularly if their family member had a dementia diagnosis and if they had struck up a relationship with another care home resident.

Care home staff participants shared examples from their practice and/or personal experiences where sexuality, intimacy and relationship needs were expressed or desired ranging from walking in on residents together, inappropriate touching by a resident on another resident and a member of staff, masturbation and formation of relationships with other residents living with and without dementia and the complex moral dilemmas faced as a result of this.

The research team identified several real-world scenarios through initial qualitative content analysis from the sticky wall exercise and explored these with care home staff and residents and their significant others during the workshop. Through discussion at this workshop, care home staff reported that, despite their being other resources in this area, they did not relate specifically to the situation of that care home staff found themselves in or that it took too long to read. Care home staff identified a lack knowledge around cultural competency in sexual and gender diversity; assessing capacity, consent and safeguarding issues in general but particularly around care home residents with living with dementia; wanting to have practical solutions when working with families to navigate through situations where two residents may form a relationship in a care home and further information and knowledge about how to manage sexuality.

### 3.4 Workshop 2: co-producing educational material

Workshop 2 lasted for between 60 and 90 min. Care home staff again identified their lack of knowledge around cultural competency in sexual and gender diversity; assessing capacity, consent and safeguarding issues in general but particularly around care home residents with dementia and how to manage complex moral dilemmas. In terms of likes and dislikes around education and training, care home staff explained that they would learn better through bite-sized training that was based on realistic scenarios and a preference for interactive learning and peer-based discussion. Care home staff also expressed a preference for accessing such training through a mobile digital platform.

### 3.5 Workshop 3: commenting on the education and training e-resource prototype

Workshop 3 lasted for ~60 min. Two prototype modules entitled “*Working with Families”* and “*Capacity, Consent and Safeguarding”* were shared with care home staff, residents and their significant others for further feedback and discussion. Each prototype module was co-designed between the research team, care home staff, residents, and their significant others. A filmed scenario had been produced and scripted by the research team, informed by scenarios provided by the workshop participants. Training resources were developed around each scenario, based on previous feedback about education and training from workshop 2. Learning was designed to provide opportunities for interactive learning in the form of questions, “*stop and think”* and “*let's explore”* options which care home staff highlighted as important in workshop 2. Additional external resources were embedded to support learning outcomes. Each module was linked to the Health and Social Care Worker Code of Conduct (Skills for Care and Skills for Health, 2013).

Care home residents and their significant others reported that the prototype appeared to highlight the areas that they felt needed to be addressed in care home staff education and training in this area but did not feel able to comment on the educational style of the resource. Care home residents' and significant others felt that the filmed scenarios represented their experiences in this area, thus supporting the educational content provided to enable care home staff to meet their and their care residents needs in this area.

### 3.6 Workshop 4: staff engagement with education and training e-resource

Workshop 4 lasted for ~60 min. The final version of the interactive education and training e-resource was shared with care home staff using a laptop and projector. Six modules were developed based on participants priority areas: (i) Working with Families; (ii) Capacity, Consent and Safeguarding; (iii) Sex and Gender Diversity; (iv) Complexity; (v) Expressing and Managing Sexuality and (vi) Communication (Table 4). Each module took around 30-min to complete and included watching video clips of case studies—for further details see Horne et al. (2022). Care home staff were then invited to navigate through the e-resource. Care staff liked the look, feel and content of the education and training e-resource, particularly the quizzes, but not necessary the assessment measures used. However, in general care staff expressed that it was something they needed and that they would like to undertake. Some care home managers felt the training could be facilitated by their training lead in groups to generate peer-based discussion around the scenarios provided within the modules whilst smaller care homes liked that the e-resource was web-based so that care staff could access this on an individual basis as and when. All care home staff stated that the education and training e-resource would be useful to their practice and professional development.

**Table 4:** Final education and training package modules.

**Module**	**Title**	**Content**
1	Working with families	This module considers the Mental Capacity Act, safeguarding and potential practical solutions when working with families as they navigate through a situation where two residents strike up a relationship in a care home.
2	Capacity, consent and safeguarding	This module focuses on the importance of capacity and consent as the relationship of two residents, both of whom are living with Alzheimer's Disease, becomes increasingly intimate.
3	Sex and gender diversity	This module explores some of the challenges which members of the LGBTI+ community face when engaging with health and social care services.
4	Complexity	This module follows an older resident who has a Learning Disability and dementia as staff help him to understand the roles of the staff and the organization amid the residents misunderstanding and sexualized behavior.
5	Expressing and managing sexuality	This module focuses on the fictional voice of care home staff as they talk about their perceptions and feelings regarding residents' sexualized behavior and how this impacts on their caregiving.
6	Communication	Recognizing the need for practical guidance, this module offers advice on effective communication around sensitive topics and how developing good reflective practice can help staff to think through issues relating to the sexual, intimacy and relationship needs of the residents they care for.

The finalized modules (Table 4) were situated on an OpenEdu (now ATHENA) platform at the University of Leeds.

## 4 Discussion

This methodology paper aimed to present the CBPR approach undertaken to create and develop an education and training e-resource designed to facilitate care home staff to support the sexuality, intimacy and relationship needs of older care home residents. Using CBPR to co-design an education and training e-resource was an innovative way of engaging a diverse range of participants in this sensitive research area to develop a more user centered e-resource. Although collaborative research approaches, such as co-design are now fairly established within care home research and practice with people living with and without dementia (Nevay and Lim, 2015; Rodgers, 2018) and in dementia-specific education and training program for home care workers (Goh et al., 2022), co-design with regards to developing education and training resources in sensitive topic areas to improve staff attitudes and knowledge about care delivery and management remains relatively new in care home environments (Horne et al., 2021).

Using CBPR to co-produce the education and training e-resource informed the real-world examples and the units of learning which resulted from discussions with care home staff, residents and their significant others. One particular example, to highlight the benefits of using such an approach, concerns initial feedback from participants about the dialogue and language used within the scenarios, expressing the view that “*carers would not say/do that!”*. This was a valuable insight which enhanced subsequent development of the scenarios as the research team responded by discussing the feedback with the actors. As a result, the dialogue was changed, and actors were asked to ad-lib a little more to bring a sense of authenticity to the scenarios.

As in other studies conducted in care homes (Goodman et al., 2011), the culture, organization and structure of care delivery within residential care settings required the research team to not only consider care home residents health-related limitations, but also their capacity to consent. Therefore, many of the residents were purposely selected by the care home managers based on those who had expressed a wish to have an intimate relationship or who were known to exhibit sexualized behavior. However, the number and diversity of care home residents invited to research studies may be limited by using this approach which could potentially lead to ethical and methodological problems associated with selection bias and under recruitment (Goodman et al., 2011; McMurdo et al., 2011). Although CBPR offers a framework for researching sensitive subject areas with vulnerable groups, research teams need to develop more context-relevant approaches to engaging community members in care homes that recognizes the ways that care home culture, organizational context and the capacity to consent mediate the research process as well as successful implementation of research initiatives (Bunn et al., 2020).

Working with care homes and discussing the tensions and agendas of each participant group enabled the co-creation of an education and training e-resource which was informed and rooted in everyday experience and practice. The resulting online, six module education and training e-resource was designed in a format which is accessible, informed, relevant to practice, engaging and interactive; indicative of the preferred training choices of the care home staff who participated. Co-production enabled the identification of real-world situations, an understanding of the knowledge gaps and the subsequent development of an e-resource that was pitched at an appropriate level using accessible language. The multi-disciplinary nature of the advisory group ensured that the resources were mapped to everyday practice and included relevant elements for practice—safeguarding, law, informal and formal carer voice, disability awareness, and the project teams' expertise in teaching and education ensured that the education and training e-resource was pedagogically sound.

Care homes can be complex, unpredictable environments (Dudman et al., 2018), so the use of CBPR was a useful approach to engaging with care home staff, residents, and their significant others as the approach was found to be adaptable and flexible in addressing the needs of care home staff, residents, and their significant others in contributing to and participating in the development of an educational e-resource. However, using CBPR in the care home environment did present some challenges. For example, the need for extra time for recruiting and engaging with care homes, care home staff, care home residents and their significant others, navigating the various tensions and respecting the various stakeholders' viewpoints could occasionally be demanding. However, developing good professional relationships with care home managers early in the study, grouping care home managers and care home staff, care home residents and their significant others in separate workshops, assisted in managing complaints (from care home residents and significant others) and competing demands to facilitate achieving the agreed goals for the research study. This approach was used as a response to our findings from a previous study about researching sexuality, intimacy and relationship needs in aged care facilities (Simpson et al., 2017a). This experience is similar to and builds on previous research that have used such an approach in developing health interventions and training resources (Kirk et al., 2021; Goh et al., 2022).

Recruiting from small, medium and large care homes, in both the private and public sector, enabled the research team to observe the diversity of care home environments in terms of undertaking co-design approaches. There were a range of places within the participating care homes to conduct the workshops, from the resident dining area to a large training room. In some care homes, presentation facilities were available, in others they were not.

Care home staff attendance was generally consistent across the four workshops which allowed for an in-depth understanding of care home staff views and experience, responding to Thys et al.'s (2019) call for greater open communication toward sexuality, intimacy and relationship needs in residential care settings. This was evident in the current study as it became apparent that care home staff were keen to recount situations they had experienced in practice and openly discussed the ethical and moral dilemmas that they faced in a care home context which strived to provide person-centered care whilst attempting to navigate family concerns and wishes. The findings of this study build on and inform the existing evidence of nuance and complexity when supporting older residents', with and without dementia, sexuality, intimacy and relationship needs (Youell et al., 2016). A limitation of this study was care home staff recruitment—all participants identified themselves as white British, which is not reflective of the care home workforce (The Migration Observatory, 2023). Future research should seek out the barriers to the recruitment of care home workers from minority backgrounds in research around sexuality, intimacy and relationship needs among older care home residents.

Care home routines presented occasional tensions between a desire to keep to the project schedule and participants' availability. For example, an appointment had been made with a resident via the care home manager, for an interview which then needed to be rescheduled as this clashed with a regular dominoes session. However, these issues were navigated with good humor, which added to the relationships built up between the research team and the care homes. An emphasis on respecting and understanding the schedules of each participant and gratitude for their participation helped to mitigate any difficulties. This approach is supported by previous research undertaken in care homes around recruitment and retention (Goodman et al., 2011; Meekes et al., 2021).

Within each facilitated workshop, a sense of collaboration, engagement and co-production was engendered by the research team assuring care home staff, care home residents and their significant others that they were “*experts by experience”*; that their involvement was a valuable contribution to knowledge (Brett et al., 2010; Elg et al., 2012; Pols, 2014) and ensuring that the education and training e-resource was informed and rooted in everyday experience and practice. Engagement of all participants was encouraged, with facilitators gently prompting those who were less confident by asking people to take a turn to speak on a subject without interruption or comment from other people, allowing people to pass a turn, providing printed questions for them to answer and the use of post-it notes. Having a range of resources available, pens, paper, etc. meant that participants could choose a medium to communicate that better suited them. Using post-it notes and other creative resources in design has been found to facilitate shared understanding during ideation activities (Warr and O'Neill, 2007; Harboe and Huang, 2015). However, for care home residents with mobility issues and space afforded within the care home, the sticky wall method was not practical. It also became clear that some care home staff were anxious about making notes which gave a sense of the differing educational levels within the workforce. Noting some reticence to participate, the research team suggested that participants discuss their experiences and offered to take notes as the discussion evolved and post post-it notes on the storyboard on their behalf. This offered an alternative option for those who were reluctant to write notes but enabled engagement through discussion, confirmation of refinement of notes made on their behalf. Where possible, two members of the research team facilitated the sessions to enable one to facilitate the workshop and the other to observe group interaction. This also allowed for a degree of flexibility within the workshops and enabled greater collaboration between participants, as well as providing for a more sensitive style of questioning and communicating with participants. This was a recommendation highlighted by participants in our previous work on researching intimacy and sexuality in aged care facilities (Simpson et al., 2017a). Future research needs to consider flexible approaches to data collection that incorporates cultural and generational experiences.

A benefit of using a CBPR approach in this study was the sharing of real-world situations and experiences by care home staff, residents, and their significant others. One case in point was an account of a resident who had multiple challenges including learning disability and dementia. The resident had previously lived in the community, with his mother, who had recently died. As a result, he was moved into a care home as living in the community alone was no longer possible. This resident perceived that the care home staff were sex workers and regularly asked for sexual encounters. On presenting this situation to the broader research team, this example was felt to be an unusual case scenario and, therefore, less credible to build a learning point around. However, the safeguarding lead, who was a part of the advisory board, argued that this was not an uncommon situation. As such, an adaption of this scenario was the basis for the module entitled “*Complexity”*. Without a collaborative, co-creationist approach, this example may very well not have been included.

CBPR is known to work well when relationships between academic groups and communities are congenial (Coombe et al., 2020). Within the current study, participant retention was linked with care home management commitment to the study and knowledge of previous research undertaken. In care homes where they had previously taken part in research or had management level employees who had research experience, attendance at the four workshops was higher than where managers were less involved. One of the aims of CBPR approach is to bring about positive change (Jensen and Laurie, 2016), as a consequence of attending the four workshops, care home staff at three of the participating care homes reported that discussions around meeting the sexuality, intimacy and relationship needs of their residents had taken place in the periods of time before and after the workshop. One care home was actively seeking ways in which to develop a sexuality, intimacy and relationship needs policy which was being discussed with other care homes within the group. CBPR approach principles would suggest that the implications of taking part in a study can result in unexpectedly positive benefits for the community and create system changes and new unanticipated projects and activities (Jagosh et al., 2012).

Whilst care home staff were invited to take part in four workshops, care home residents and their significant others were invited to two of the workshops—workshops one and three. As participants required capacity to consent, care home residents were recruited via care home managers who knew their residents well. As a result, many of the residents were purposely selected based on the care home managers knowledge of residents who had expressed a wish to have an intimate relationship or who were known to exhibit sexualized behavior. This limited the care home resident involvement in the research study to just those who were overt in their relational need and expression. Consequently, a limitation of the study was the low numbers of residents who participated in the study and the under representation of those diagnosed with dementia and from the LGBTQ+ community. However, insights into the accounts of dementia and intimacy of care home residents who were unable to participate where provided through their family member and/or significant other, as well as care home staff.

The literature acknowledges that using gatekeepers to recruit care home residents may result in the gatekeeper potentially denying the right to decide whether people with and without dementia should participate in research and be based on the gatekeeper's judgement about who should be involved (Bartlett and Martin, 2002; Sherratt et al., 2007; Goodman et al., 2011). However, using gatekeepers does safeguard care home residents' rights and the involvement of caregivers, particularly with vulnerable groups, safeguards the rights of care home residents living with and without dementia (Chandra et al., 2021). Using CBPR as an approach minimized the potential distress to care home resident engagement in the study based on the belief that care home managers knew their residents well and would be aware of who was able and willing to participate. CBPR approaches acknowledge the communities' strengths, including local and institutional knowledge, for example gatekeepers, communication styles and skills, community engagement and relationship building (Collins et al., 2018).

Adaptation to the way the workshops were organized and conducted was needed, as a group format was not conducive for care home resident discussion. Through discussion with care home residents, one-to-one discussions were reported to be much preferable for care home residents to be involved with the research study. Care home residents spoke generally about the importance of relational wellbeing including sex and intimacy but did not specifically refer to their own experiences but those of “*others”*. In the main, care home resident accounts informed the “*expressing and managing sexuality and intimacy”* module within the research study and contributed to the authenticity of the language used. Care home residents accounts around sexuality, intimacy and relationship needs expressed an understanding that sex would not be allowed now that they lived in a care home.

One particular challenge encountered within the care home resident one-to-one discussions was that of a disclosure which resulted in the need to raise a safeguarding concern. The disclosure did not relate to the participant, but to another resident and was of sufficient concern to follow the research protocol and report to the appropriate authorities. This highlighted ethical concerns around capacity to consent to sexual intimacy, the formation of new relationships and vulnerability to sexual abuse in care settings, which has been highlighted in previous work in this area (Burgess and Phillips, 2006; Tarzia et al., 2012; Wiskerke and Manthorpe, 2016).

Conducting and facilitating the significant other workshops was challenging as the family carers generally had their own agenda for attending the workshops despite being fully informed of the nature and scope of the research study through the consenting process. Most saw the workshop as an opportunity to raise general issues about the levels of care their loved ones were receiving in the care home. Drawing the discussion back to the study research questions, one participant disclosed that her mother had been a victim of sexual assault by another care home resident. This was clearly upsetting for the participant and the distress protocol was instigated. The participant chose not to attend future workshops. Nonetheless, significant others' accounts informed the “*working with families”* and “*expressing and managing sexuality and intimacy”* modules for the e-resource and contributed to the authenticity of the language used. During workshop discussions it was evident that family carers wanted their loved one to be safe above all else. These participants also wanted their loved ones to be happy and expressed the view that intimate behaviors, such as hugging, holding hands, and kissing on the cheek were acceptable but that sex was not. Using CBPR approach with significant others allowed for a more sensitive approach to taking part and being involved in research around sexuality, intimacy and relationship needs of older care home residents and assisted in the co-design process of developing the e-resource. The small sample size of significant others recruited is a limitation. Future research should seek out the barriers to the recruitment of family, carers and significant others in research around sexuality, intimacy and relationship needs among older care home residents.

## 5 Conclusion

CBPR was a useful approach to engaging and working collaboratively with care home staff, residents and their significant others on the issues that mattered to them. Previous work with care home residents and staff contributed to the aims and objectives of this study. Co-production employed within CBPR enabled the research team to work with and feedback to participants within the current study, allowed discussion to take place between workshops and verify or amend the themes the research team had identified from previous workshops. In some care homes there was limited engagement, which identifies the challenges in using this approach. However, using CBPR enabled care home staff, residents, and family members to contribute to the authenticity of the language used in the resource, directed the actions taken, and ensured that the education and training resource reflected real-world experiences which may not have otherwise been captured. This study has demonstrated how care home environments are responsive to CBPR methodology. Within this research study partial inclusion of residents was gained. Future studies might wish to consider an additional workshop or one-to-one conversations with older residents and their significant others around the use of CBPR so that these participants feel comfortable and familiar with the methodology as well as the research aims.

## Data availability statement

The raw data supporting the conclusions of this article will be made available by the authors, without undue reservation.

## Ethics statement

The studies involving humans were approved by School of Healthcare Research Ethics Committee, University of Leeds. The studies were conducted in accordance with the local legislation and institutional requirements. The participants provided their written informed consent to participate in this study.

## Author contributions

MH and JY conducted investigation, managed the data, conducted data analysis, drafted the manuscript, handled project administration, and wrote the first draft of the manuscript. CB, LB, PS, and TD wrote sections of the manuscript and approved the submitted version. All authors contributed to conception, design of the study, and final data analysis.
